# Effects of maternal and fetal *LEP* common variants on maternal glycemic traits in pregnancy

**DOI:** 10.1038/s41598-017-18117-z

**Published:** 2017-12-18

**Authors:** Rong Lin, Hongfang Ju, Ziyu Yuan, Caicai Zhang, Liangliang Zeng, Yuantian Sun, Zhenyu Su, Li Jin

**Affiliations:** 1 Department of Biology, Hainan Medical College, Haikou, Hainan China; 2 Department of Gynaecology and Obstetrics, Taizhou People’s Hospital, Taizhou, Jiangsu China; 3 Fudan-Taizhou Institute of Health Sciences, Taizhou, Jiangsu China; 4 Ministry of Education Key Laboratory of Contemporary Anthropology and State Key Laboratory of Genetic Engineering, Collaborative Innovation Center for Genetics and Development, School of Life Sciences, Fudan University, Shanghai, China; 5 Department of Physiology, Hainan Medical College, Haikou, Hainan China; 6 CAS-MPG Partner Institute for Computational Biology, Shanghai Institute for Biological Sciences, Chinese Academy of Sciences, Shanghai, China

## Abstract

Previous studies suggest that leptin (LEP) has an important role in glucose metabolism in the nonpregnant state. During pregnancy, circulating maternal concentrations of leptin rise significantly, mainly due to increased secretion of leptin from maternal adipose tissue and placenta. This study aimed to analyze the impact of maternal and fetal common *LEP* variants on glucose homeostasis in the pregnant state. Several glycemic traits, including fasting plasma glucose, fasting plasma insulin (FPI), and plasma glucose 1 hour after a 50-g oral glucose load, were measured in 1,112 unrelated Chinese Han pregnant women at 24–28 weeks gestation. Homeostatic model assessment (HOMA) was used to assess beta cell function (HOMA1-β and HOMA2-β) and insulin resistance (HOMA1-IR and HOMA2-IR).The relationships between glycemic traits and 12 *LEP* variants were determined. After applying the Bonferroni correction, we detected that (1) maternal rs10954173 and fetal rs10244329 were associated with maternal FPI although the effect of fetal rs10244329 may be not independent of maternal rs10244329, and (2) maternal rs12537573 was associated with maternal FPI and HOMA2-IR. This study provides genetic evidence that both maternal and fetal *LEP* polymorphisms may affect maternal glucose metabolism in pregnancy.

## Introduction

Increasing maternal glucose concentrations during pregnancy are correlated with adverse pregnancy outcomes including birth weight above the 90th percentile, primary cesarean delivery, neonatal hypoglycemia, and fetal hyperinsulinemia^1^. These correlations are continuous over the entire range of maternal glucose concentrations, even among those concentrations below the diagnostic cut points for gestational diabetes mellitus (GDM). No specific concentration, at which elevated maternal glucose concentrations became clinically important, has been identified. Gestational glycemic traits and GDM are considered to result from the complex interplay of both genetic and environmental factors^2,3^. A population-based study has yielded heritability estimates ranging between 30 and 71% for fasting plasma glucose (FPG) concentrations during pregnancy^3^.

Leptin, a circulating hormone, is secreted abundantly from adipose tissue. It regulates metabolism and energy homeostasis^4^. Several studies have shown that leptin can modulate insulin secretion^5^. Common variants in the human leptin (*LEP*) gene have been demonstrated to associate with leptin concentrations^6–8^, fasting serum insulin concentration^9^, insulin resistance^9^ and type 2 diabetes mellitus (T2DM)^9^. Given the overlap in genes associated with glycemic traits in gravid and nongravid populations, it may be hypothesized that maternal *LEP* variants may be associated with maternal glucose concentration or/and related traits in pregnancy.

Recent reports have suggested that leptin may play an important role in the biology of pregnancy^10^. During pregnancy, circulating maternal concentrations of leptin rise to maximal values in the second trimester, plateau in the third trimester and drop below pre-pregnancy concentrations around delivery^11,12^. The main source of increased maternal leptin secretion during pregnancy could be maternal adipose tissue and placenta. That is to say, leptin from the placenta also contributes to the rise in maternal concentrations of leptin. Leptin is also produced by nonadipose tissue, namely, placental cytotrophoblasts and syncytiotrophoblasts, villous vascular endothelial cells, and amnion cells from uteri of pregnant women^13,14^. Pregnant women secrete a considerable amount of leptin from the placenta into the maternal circulation as compared with nonpregnant obese women^13^. The placenta might be capable of producing equal or even higher concentrations of leptin than adipose tissue^15,16^. Human *LEP* gene has a placental specific upstream enhancer^17^, implying that, in human, placental *LEP* is differentially regulated when compared to *LEP* of adipose origin.

An investigation on leptin expression in third trimester placenta showed that abnormal pregnancies complicated with maternal insulin-dependent diabetes or GDM exhibited higher concentrations of placental leptin mRNA and protein relative to normal pregnancies^18^. Leptin expression increases in placentas from GDM^19^. Plasma leptin was higher in pregnant women with GDM than in pregnant women with normal glucose tolerance and increased in both groups when compared with the non-pregnant women^20,21^. GDM is a pathology characterized by increased placental leptin contributions to enhanced maternal leptin concentrations. Thus we hypothesized that placental variants in the human *LEP* gene might be related to maternal glucose concentration or related traits in pregnancy. The umbilical cord blood, fetus, placental cytotrophoblasts and syncytiotrophoblasts, villous vascular endothelial cells, and amnion cells from uteri of pregnant women have the same DNA. In this study, the DNA extracted from umbilical cord blood was used to represent the DNA of the placenta and fetus.

In the present study, we investigated the distribution of common *LEP* variants in Chinese pregnant women and their fetuses. Then, we compared maternal glucose concentrations and related traits between different maternal and fetal genotypes to identify whether these maternal and fetal variants are associated with differences in glucose metabolism in Chinese pregnant women. The objective of this study was to investigate the influence of maternal and fetal common variants in the *LEP* gene on plasma glucose, insulin values, and insulin resistance in the fasted state among pregnant women.

## Results

**Figure 1:**
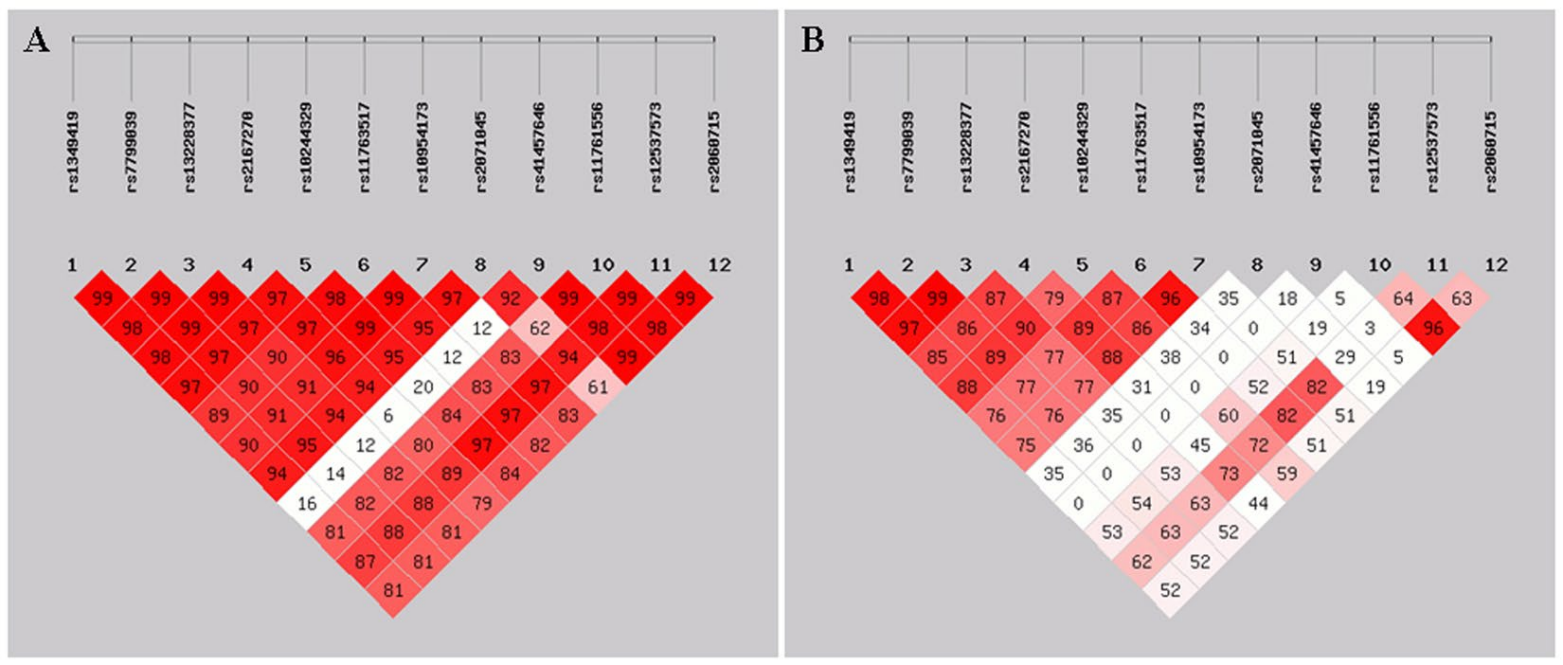
Linkage disequilibrium (LD) plot of the *LEP* locus in all pregnant subjects. This Figure shows LD values D’ (**A**) and *r* ^2^ (**B**) between each SNP. Each diamond contains the LD value between the two SNPs that face each of the upper sides of the diamond, ex: *r* ^2^ between rs1349419 (SNP 1) and rs2167270 (SNP 4) is 0.85; the redder the diamond, the higher the LD value. (SHEsis Software, ver. online)^39^

Strong pair-wise linkage disequilibrium (LD) was observed between the single nucleotide polymorphisms (SNPs) (each D’ > 0.80) except between SNP 9 and SNP 1–7, between SNP 8 and 10, between SNP 8 and 12, as well as between SNP 4 and 12 (Fig. 1A). The values of pair-wise *r* ^2^ between SNP 1, 2, 3 and 4 were all greater than 0.80 which indicated that SNP 1, 2, 3 and 4 were highly linked with each other. The values of pair-wise *r* ^2^ (1) between SNP 5, 6 and 7, (2) between SNP 6, 7 and 11, as well as (3) between SNP 10 and 12 were all also greater than 0.80 (Fig. 1B). Therefore, we arbitrarily selected *P* < 0.05/5 = 0.01 for *LEP* as nominally significant since it corresponds to a corrected *P*-value of 0.05 after correction for 5 independent SNPs in the 12 SNPs analyzed in the study. SNP 1–7 were adjacent and in LD with each pair-wise *r* ^2^ greater than 0.75. Therefore, SNP 1–7 were grouped together and designated as Block 1. We examined the associations between each genotyped SNP and glycemic traits and presented only the results that were statistically significant at the 0.05 level in Supplementary Table S1 and those statistically significant at the 0.01 level in Table 1.

**Table 1:** Relationship between *LEP* common variants and glycemic traits. Values are shown only for polymorphisms with *P* < 0.01 in analysis of variance.

		Genotype	N	Geometric mean(95% confidence interval)	*P*	*P* ^a^	*P* ^b^	*P* ^c^
24–28-week maternal fasting plasma insulin, pmol/l								
SNP 7	rs10954173_M	GG	550	51.69(49.42–54.07)	0.008	0.011	0.009*	0.008*
		GA	288	55.36(51.72–59.25)				
		AA	39	41.66(34.77–49.92)				
SNP 11	rs12537573_M	AA	577	51.45(49.24–53.77)	0.006	0.004	0.003*	0.004*
		AG	274	55.80(52.02–59.87)				
		GG	27	40.25(32.91–49.22)				
SNP 5	rs10244329_F	AA	364	54.27(51.06–57.68)	0.006	0.008	0.087^#^	0.098^#^
		AT	257	50.70(47.17–54.49)				
		TT	35	39.21(32.45–47.38)				
HOMA2-IR								
SNP 11	rs12537573_M	AA	554	0.97(0.93–1.00)	0.004	0.004	1.40 × 10^−4^*	1.75 × 10^−4^*
		AG	264	1.01(0.96–1.07)				
		GG	26	0.74(0.61–0.90)				

^a^Adjusted for maternal age at delivery, newborn sex, prepregnancy gravidity and prepregnancy parity by analysis of covariance. ^b^Adjusted for corresponding maternal or fetal variants by analysis of covariance. Adjustments for corresponding fetal variants were marked with * and adjustments for corresponding maternal variants were marked with #. ^c^Adjusted for corresponding maternal or fetal variants, maternal age at delivery, newborn sex, prepregnancy gravidity and prepregnancy parity by analysis of covariance. M: maternal genotypes, F: fetal genotypes.

### Association with 24–28-week maternal fasting plasma glucose

Neither maternal nor fetal *LEP* SNPs were associated with 24–28-week maternal FPG (data not shown). None of maternal-fetal *LEP* genotype combinations showed association with 24–28-week maternal FPG.

### Association with 24–28-week maternal plasma glucose 1 hour after the consumption of a 50-g oral glucose load

Among the 12 *LEP* SNPs, 5 maternal SNPs (rs1349419, rs7799039, rs13228377, rs11761556 and rs2060715) were associated with 24–28-week maternal plasma glucose 1 hour after a 50-g oral glucose load at *P* < 0.05 (Supplementary Table S1): homozygotes of the minor allele had significantly higher mean glucose concentrations than heterozygotes and homozygotes of the major allele. Among the 5 associated SNPs, rs1349419, rs7799039 and rs13228377 were all in Block 1. SNP rs11761556 in 3′-untranslated region (UTR) and rs2060715 in the 3′-flanking region were in high LD (*r* ^2^ = 0.96).

One fetal SNP (rs41457646) in 3′-UTR was associated with 24–28-week maternal plasma glucose 1 hour after a 50-g oral glucose load (*P* = 0.019). Maternal-fetal genotype combination of SNP rs41457646 was also associated with 24–28-week maternal plasma glucose 1 hour after a 50-g oral glucose load (*P* = 0.029), but lost its significance after adjustment for fetal SNP rs41457646 (*P* = 0.213), indicating that this association might be caused by the effect of fetal SNP rs41457646.

No significant associations were identified at *P* < 0.01. That is to say, all aforementioned associations did not pass Bonferroni correction.

### Association with 24–28-week maternal fasting plasma insulin (FPI)

Six maternal SNPs (rs1349419, rs2167270, rs10244329, rs11763517, rs10954173 and rs12537573) were associated with 24–28-week maternal FPI at *P* < 0.05 before adjustment (Supplementary Table S1): for each SNP, subjects homozygous for the minor allele had lower 24–28-week FPI concentrations compared with those carrying the major allele. Of note, 5 associated SNPs (rs1349419, rs2167270, rs10244329, rs11763517 and rs10954173) were all in Block 1, whereas rs12537573 is in the 3′-flanking region of *LEP*.

Six fetal *LEP* SNPs (rs1349419, rs7799039, rs13228377, rs10244329, rs11763517 and rs10954173) in Block 1 were all associated with 24–28-week maternal FPI at *P* < 0.05: subjects having newborns homozygous for the minor allele had lower 24–28-week FPI concentrations compared with those having newborns carrying the major allele.

Maternal-fetal genotype combinations of six SNPs (rs1349419, rs7799039, rs10244329, rs11763517, rs10954173 and rs12537573) were associated with 24–28-week maternal FPI at *P* < 0.05, but lost its significance after adjustment for corresponding maternal or fetal SNPs (all *P* > 0.05), suggesting that these associations might be caused by the effects of maternal or fetal SNPs.

If we apply a Bonferroni correction (*P* < 0.01), maternal SNP rs10954173 (in Block 1), maternal SNP rs12537573 (in the 3′-flanking region) and fetal SNP rs10244329 (also in Block 1) remained associated with 24–28-week maternal FPI (*P* = 0.008, 0.006 and 0.006, respectively) (Table 1).

### Association with 24–28-week maternal homeostasis model assessment (HOMA) β cell function index (HOMA-β)

Only two fetal *LEP* SNPs both in Block 1 (rs10244329 and rs10954173) were associated with 24–28-week maternal HOMA2-β at *P* < 0.05 before adjustment (Supplementary Table S1): subjects having newborns homozygous for the minor allele had lower 24–28-week HOMA2-β compared with those having newborns carrying the major allele. However, both associations did not pass the Bonferroni threshold of *P* < 0.01.

### Association with 24–28-week maternal HOMA insulin resistance index (HOMA-IR)

Three maternal *LEP* SNPs (rs11763517 and rs10954173 in Block 1 as well as rs12537573 in the 3′-flanking region) were associated with 24–28-week maternal HOMA1-IR at *P* < 0.05 (Supplementary Table S1): homozygous carriers of the minor allele had lower 24–28-week HOMA1-IR compared with carriers of the major allele. Similarly, the 3 maternal SNPs and another maternal SNP (rs2167270 in Block 1) were associated with 24–28-week maternal HOMA2-IR at *P* < 0.05 before adjustment: homozygous carriers of the minor allele had lower 24–28-week HOMA2-IR compared with carriers of the major allele. Two fetal *LEP* SNPs in Block 1 (rs10244329 and rs10954173) were associated with 24–28-week maternal HOMA1-IR at *P* < 0.05 before adjustment: subjects delivering newborns homozygous for the minor allele had lower 24–28-week HOMA1-IR compared with those delivering newborns with the major allele. Similarly, the 2 fetal SNPs and another fetal SNP (rs13228377 in Block 1) were associated with 24–28-week maternal HOMA2-IR at *P* < 0.05 before adjustment: subjects delivering newborns homozygous for the minor allele had lower 24–28-week HOMA2-IR compared with those delivering newborns with the major allele. Maternal and fetal genotype combinations of 2 SNPs (rs10244329 and rs10954173) were associated with 24–28-week maternal HOMA1-IR, and maternal and fetal genotype combinations of 4 SNPs (rs10244329, rs11763517, rs10954173 and rs12537573) were associated with 24–28-week maternal HOMA2-IR, but lost its significance after adjustment for corresponding maternal or fetal SNPs (all *P* > 0.05). With the use of a Bonferroni correction, only the association between maternal SNP rs12537573 and 24–28-week maternal HOMA2-IR again reaches significance (*P* = 0.004) (Table 1).

To summarize, at *P* < 0.05, we observed that (1) several maternal SNPs in Block 1 were associated with 24–28-week maternal plasma glucose 1 hour after a 50-g oral glucose load, FPI, HOMA1-IR and HOMA2-IR, and (2) several fetal SNPs in Block 1 were associated with 24–28-week maternal FPI, HOMA2-β, HOMA1-IR and HOMA2-IR, suggesting that (1) there may be one or more functional variants in Block 1 which affect maternal glucose metabolism in pregnancy, and (2) both maternal and fetal *LEP* gene polymorphisms may affect maternal glucose metabolism during pregnancy.

In addition, at *P* < 0.05, we detected that (1) maternal SNP rs11761556 (in 3′-UTR) and rs2060715 (in the 3′-flanking region) were in high LD (*r* ^2^ = 0.96) and related to 24–28-week maternal plasma glucose 1 hour after a 50-g oral glucose load, (2) fetal SNP rs41457646 (in 3′-UTR) was also related to 24–28-week maternal plasma glucose 1 hour after a 50-g oral glucose load, and (3) maternal SNP rs12537573 (in the 3′-flanking region) was associated with 24–28-week maternal FPI, HOMA1-IR and HOMA2-IR.

However, applying a cut off for the Bonferroni-corrected *P*-values of 0.01, we only observed that (1) maternal SNP rs10954173 (in Block 1) and fetal SNP rs10244329 (also in Block 1) were associated with 24–28-week maternal FPI, (2) maternal SNP rs12537573 (in the 3′-flanking region) was associated with 24–28-week maternal FPI and HOMA2-IR.

All four associations identified at *P* < 0.01 remained significant after controlling maternal age at delivery, newborn sex, prepregnancy gravidity and prepregnancy parity (Table 1). But when adjusted for corresponding maternal or fetal genotypes, only three associations remained significant and the association between fetal rs10244329 and 24–28-week maternal FPI lost significance (*P* = 0.087). One explanation is that the effect of fetal rs10244329 may be not independent of maternal rs10244329. As Supplementary Table S2 shown, among mothers delivering newborns with AT genotype of rs10244329, those with TT genotype not AA or AT genotype showed significantly lower FPI compared to mothers delivering newborns with AA genotype.

We also explored the associations of maternal and fetal *LEP* variants with newborn birth weight but did not find any statistically significant association (data not shown).

## Discussion

In the current study, we analyzed the association of 12 common *LEP* variants with maternal glycemic traits in Chinese Han mothers and their newborns. We could demonstrate that (1) maternal SNP rs10954173 (in Block 1) and fetal SNP rs10244329 (also in Block 1) were associated with 24–28-week maternal FPI, although the effect of fetal rs10244329 may be not independent of maternal rs10244329, and (2) maternal SNP rs12537573 (in the 3′-flanking region) was associated with 24–28-week maternal FPI and HOMA2-IR, after Bonferroni correction. These results extended previous findings that maternal genetic factors contribute to glucose metabolism during pregnancy. To our knowledge, there have been few assessments of associations between fetal variants as well as maternal-fetal genotype combinations and gestational glycemic traits. We detected that one fetal SNP was associated with 24–28-week maternal FPI. However, we did not detect any significant associations between maternal-fetal genotype combinations and maternal glycemic traits.

The current study identified *LEP* variants associated with 24–28-week maternal FPI and HOMA2-IR after Bonferroni correction. Administration of leptin antagonists to normal mice increases plasma insulin levels and promotes insulin resistance^22^. Leptin inhibits insulin secretion and insulin gene expression. In turn, insulin stimulates leptin synthesis and secretion^5,23,24^. This is a hormonal regulatory feedback loop. When compared to the nonpregnant state, leptin is 2–3 fold higher with a peak at around 28 weeks of gestation^25^, which may visibly affect FPI and HOMA-IR around 28 weeks of gestation.

Hoffstedt *et al*.^26^ examined whether SNP 2 (rs7799039, also named G-2548A) influences leptin expression. Adipose tissue leptin secretion rate in subjects with the AA genotype (*n* = 11) was twice as high as in GA/GG subjects (*n* = 28). Moreover leptin mRNA levels in AA subjects (*n* = 7) were about 60% higher than in GA/GG subjects (*n* = 6). These suggested that SNP 2 or/and other variants in LD with SNP 2 may possibly affect leptin expression at the transcriptional level.

The electrophoretic mobility shift assay revealed that nuclear extracts derived from both U937 cells and human adipocytes form a protein-DNA complex with the leptin SNP 2 G/A polymorphic site and bind with higher affinity to the SNP 2 A-site^26^. This indicated that SNP 2 might indeed modify the *LEP* transcription rate, but did not exclude the possibility that other variants in LD with SNP 2 may also be regulatory polymorphic sites. Therefore, in this study, we investigated another 6 SNPs (SNP 1 and 3–7) which are in LD with SNP 2, and SNP 1-7 are all located upstream of the translation start site (Supplementary Fig. S1) and in one block designated as Block 1.

Vasku *et al*.^27^ only examined maternal SNP 2 and showed that maternal SNP 2 was related to GDM. The present study showed that (1) maternal SNP 2 was associated with maternal 50-g 1-h glucose concentrations between 24 and 28 gestational weeks, and (2) fetal SNP 2 was associated with maternal fasting insulin concentrations between 24 and 28 gestational weeks. But both associations did not pass Bonferroni correction. However, in the present study, maternal SNP 7 (rs10954173) and fetal SNP 5 (rs10244329), which both are in Block 1 and in LD with SNP 2, showed association with 24–28-week maternal FPI after Bonferroni correction. SNP 7 has been reported to be associated with T2DM^9^. SNP 7 and SNP 5 are located 632 and 3,383 bases upstream of the start codon ATG, respectively. Different associated SNPs were detected in the maternal and fetal *LEP* gene, which might be suspected due to a divergence in transcriptional regulation in placenta and maternal adipose tissue^17,28^. In human, a placental specific enhancer has been found in the *LEP* upstream^17^.

Another study investigated the effects of 15 maternal *LEP* SNPs on gestational fasting glucose in 3,836 white and 1,713 Thai pregnant women, and found no positive associations^29^. The present study revealed similar results.

SNP 4 (rs2167270, also named A19G) in 5′-UTR of *LEP* has been shown to be statistically significantly related to post-transplant diabetes mellitus^30^ and serum leptin concentrations in patients with differentiated thyroid cancer^31^. By comparison, the current study indicated that maternal SNP 4 was associated with 24–28-week maternal FPI and HOMA2-IR just at *P* < 0.05 but did not reach significance under the Bonferroni threshold.

SNP 9 (rs41457646) in 3′-UTR has been documented to be not correlated with fasting blood glucose, fasting serum insulin, HOMA1-IR in a non-gravid population^9^. Similarly, for SNP 9, none associations reached significance under the Bonferroni threshold, although fetal SNP 9 showed an association with 24–28-week maternal plasma glucose 1 hour after a 50-g oral glucose load at *P* < 0.05 in the current study.

SNP 10 (rs11761556) in 3′-UTR was identified to be associated with T2DM^9^. However, in the present study, only maternal SNP 10 was detected to be associated with 24–28-week maternal plasma glucose 1 hour after a 50-g oral glucose load at *P* < 0.05 but significance was lost after Bonferroni correction.

The allelic frequencies of the 12 *LEP* variants in the present study and East Asians were similar, but most of them are apparently distinct from those in other populations (Europeans, Africans, Ad Mixed Americans and South Asians) (available from the 1000 Genomes Project, based on GRCh38)^32^ (Supplementary Table S3). For example, except for SNP 9 (rs41457646), the allelic frequencies of other SNPs in East Asians and Europeans are obviously different. Genetic differences among populations may lead to some disparities of associations between genetic variation and complex traits among populations.

One limitation of our study is the relatively modest sample size. For most SNPs, the number of the genotype combination where both pregnant women and their children were homozygous for the minor allele was small, usually less than 10. So the analyses may not have enough statistical power to detect the associations between maternal glycemic traits and maternal-fetal gene combinations. One reason for this is the practical difficulties in collecting samples from mothers and their fetuses rather than just one. In the present study, only 513 mother-fetus pairs were recruited. We did not successfully collect umbilical cord blood samples for 416 mothers’ neonates and venous blood samples for 183 neonates’ mothers. Thus, replication of these associations in larger populations of pregnant women and their newborns will be needed to elucidate these results. Furthermore, how maternal and fetal genes interact is complex, and the interactions between different genes may be different. A simple additive effect is only one of them and so the analysis for maternal-fetal genotype combinations is just a preliminary attempt in our study. More mother-fetus pairs are required for deeper analyses.

Another limitation of our study is absence of functional evidence. Therefore, the significant SNPs are not eventually identified as the true causal variant. More comprehensive efforts and experimental studies are required to find the potential functional significance of SNP rs10954173, rs10244329 and rs12537573 or other variants linked to them in glucose metabolism in pregnancy.

In conclusion, our study shows that both maternal and fetal *LEP* SNPs are associated with maternal glycemic traits during pregnancy. This suggests that both maternal and fetal genes may affect maternal glucose metabolism. Several maternal common genetic variants have been associated with GDM or gestational glycemic traits, but little is known about how fetal genetic variation influences GDM or maternal glycemic traits through the placenta. The present study demonstrated for the first time that fetal genetic variation at *LEP* is related to maternal glycemic traits in pregnancy.

## Methods

### Study population

The study involved 1,112 unrelated Chinese Han women who delivered singleton newborns at Taizhou People’s Hospital between October 2010 and June 2013. Subjects ranged from 19 to 44 years of age and their husbands were all Chinese Han. The antenatal visits for all subjects were conducted mainly in Taizhou People’s Hospital. All women were screened for GDM between 24 and 28 gestational weeks with a 50-g 1-h glucose challenge test after overnight fasting of 8–14 hours. At the same time, fasting glucose and insulin concentrations were also determined. If the test was positive (fasting glucose ≥ 5.6 mmol/l or plasma glucose 1 hour after ingestion of 50 g sugar ≥ 7.8 mmol/l), the women underwent an oral glucose tolerance test with 75 g sugar. GDM was diagnosed according to the American Diabetes Association (ADA) guideline^33^. Plasma glucose was measured by a hexokinase method (Cobas P800, Roche Diagnostics, Mannheim, Germany). Plasma insulin was analyzed using an electrochemiluminescence immunoassay on a Cobas e601 analyzer (Roche Diagnostics, Mannheim, Germany). Nearly 94.9% of subjects had fasting glucose concentrations less than 5.6 mmol/l. No previous diagnosis of diabetes type 1 and type 2, autoimmune and inflammatory diseases, neoplasmatic diseases and chronic infections was reported in any participant. We also excluded individuals who reportedly used antidiabetic medication before the screening.

Using the fasting insulin and glucose values, pancreatic β-cell function and insulin resistance were calculated using the HOMA analysis as follows: HOMA1-β = 20 × fasting insulin (μU/ml)/[fasting glucose (mmol/l)-3.5], HOMA1-IR = fasting insulin (μU/ml) × fasting glucose (mmol/l)/22.5. For a fasting glucose concentration less than 3.5 mmol/l would preclude calculation of a HOMA1-β, HOMA1-β of 25 subjects could not be determined. HOMA2-β and HOMA2-IR were also calculated with a HOMA2 Calculator v2.2 in subjects whose plasma glucose and insulin fell into plausible ranges (glucose 3.0–25.0 mmol/l, insulin 20–400 pmol/l)^34^. HOMA2-β and HOMA2-IR values were available for 1019 (92%) out of 1,112 subjects. The higher the HOMA-β value the more the insulin the beta-cells have to secrete to handle existing blood glucose concentration, and the higher the HOMA-IR value the more the insulin resistance. For epidemiological studies, HOMA is more suitable and convenient, compared with the gold standard–more laborious euglycemic clamp^35^ or the frequently sampled intravenous glucose tolerance test^36,37^. HOMA1-IR mainly reflects hepatic insulin resistance, and HOMA2-IR reflects hepatic and peripheral insulin resistance^34^.

Basal characteristics of all subjects are given in Table 2. A few subjects had missing baseline data on fasting insulin concentrations (*n* = 51) or 50-g 1-h glucose concentrations (*n* = 53). Most of them (97.8%) delivered at 37 to 42 weeks of gestation (mean 39.4 ± 1.1 weeks) and most of their newborns (98.7%) had birth weights between 2500 and 4500 grams (mean 3400.9 ± 417.4 g).

**Table 2:** Clinical characteristics of study participants. Data are arithmetic mean ± standard deviation or percentages.

Demographic	N	
Maternal age at delivery, years	1109	26.7 ± 3.8
Paternal age at delivery, years	1106	28.1 ± 4.4
Prepregnancy gravidity	1110	0.7 ± 1.0
Prepregnancy parity	1110	0.2 ± 0.4
Prepregnancy BMI, kg/m^2^	1092	20.7 ± 2.6
24–28-week maternal fasting plasma glucose, mmol/l	1112	4.53 ± 0.61
24–28-week maternal fasting plasma insulin, pmol/l	1061	65.19 ± 75.94
24–28-week maternal plasma glucose (1 hour after the consumption of a 50-g oral glucose load), mmol/l	1059	7.11 ± 1.44
HOMA1-β	1036	233.83 ± 263.85
HOMA1-IR	1036	1.94 ± 2.62
HOMA2-β	1019	128.31 ± 48.13
HOMA2-IR	1019	1.10 ± 0.61
Gestational age at delivery, weeks	1112	39.4 ± 1.1
Newborn sex, % male	1110	51.9%
Newborn birth weight, g	1110	3400.9 ± 417.4

BMI, body mass index; HOMA-β, homeostasis model assessment-β-cell function; HOMA-IR, homeostasis model assessment-insulin resistance.

For genomic DNA isolation, the mothers’ venous blood samples were collected in the days surrounding delivery, and the neonates’ umbilical cord blood samples were collected at birth after removal of contaminants and disinfection with alcohol on the cords’ surface. Finally, a total of 1,625 blood samples (i.e. 929 maternal venous blood samples and 696 neonatal cord blood samples) were collected for genomic DNA isolation, including 513 maternal venous blood samples and 513 corresponding neonatal umbilical cord blood samples, 416 maternal venous blood samples without corresponding neonatal umbilical cord blood samples, as well as 183 neonatal umbilical cord blood samples without corresponding maternal venous blood samples. The study protocol was approved by the Ethics Committee of Hainan Medical College and the local Ethics Committee, and informed consent was obtained from each study participant. All methods were carried out in accordance with the approved guidelines.

### SNP selection and genotyping

A combined tag and candidate SNP approach was used to select SNPs in *LEP*. Eight tag SNPs across the *LEP* gene and its 5 kb up-/downstream region (chromosome 7: 128236278. 128262628 26.35 kbp, human genome reference assembly GRCh38/hg38), including rs7799039, rs2167270, rs10244329, rs11763517, rs2071045, rs41457646, rs11761556 and rs12537573, were selected on the basis of LD patterns observed in the phase III Han Chinese in Beijing (CHB) and Southern Han Chinese (CHS) populations with an *r* ^2^ of at least 0.9 (Supplementary Fig. S1 and Table S3). Only SNPs with minor allele frequency (MAF) greater than 5% were considered. In addition, three SNPs located upstream of the translation start site (rs1349419, rs13228377 and rs10954173), as well as one SNP in the 3′-flanking region (rs2060715) were selected.

These 12 SNPs were genotyped in all samples using a custom-by-design 48-Plex SNPscan^TM^ Kit (Cat#:G0104; Genesky Biotechnologies Inc., Shanghai, China), which was developed according to patented SNP genotyping technology by Genesky Biotechnologies Inc. As described by Chen *et al*.^38^, it was based on double ligation and multiplex fluorescence polymerase chain reactions. Maternal and fetal DNA samples were interspersed within 96-well plates. Each plate included a negative control and 5 blind duplicate samples. Each polymorphism was successfully assayed in ≥99.9% of the samples (Supplementary Table S3). The concordance rates were more than 99% based on 5.5% duplicate samples. Genotypes of all 12 SNPs fitted Hardy-Weinberg equilibrium (HWE) (all *P* > 0.05) (Supplementary Table S3).

### Statistical analysis

HWE for each SNP was examined using a chi-squared test. Pair-wise LD based on the D’ and *r* ^2^ statistics for the 12 SNPs was calculated by the SHEsis (http://analysis.bio-x.cn/myAnalysis.php)^39^ and SNPStats (http://bioinfo.iconcologia.net/snpstats/start.htm)^40^ softwares. The statistic D’ < 0.20 was thought to have no LD, D’ > 0.50 had a LD, D’ > 0.80 had a strong LD and D’ = 1 is in complete LD. The statistic *r* ^2^ < 0.50 was considered to have low LD, *r* ^2^ > 0.50 had a moderate high LD, *r* ^2^ > 0.80 had a high LD and *r* ^2^ = 1 is in perfect LD. Variants were considered to be independent if the pair-wise *r* ^2^ was less than 0.80. Because several SNPs tested were in high LD with each other and hence were far from independent, multiple testing adjustment was performed by a Bonferroni correction using the effective number of independent variants.

A natural log transformation was conducted for fasting insulin concentrations, HOMA1-β, HOMA1-IR, HOMA2-β and HOMA2-IR to provide an approximately normal distribution. The differences of maternal glycemic traits among genotype groups were examined by analysis of variance (ANOVA) using Statistical Package for Social Science (SPSS; Chicago, IL, USA) version 15.0. If the mother fetus pair possessed exactly three or four copies of a specific allele, this combination may increase risk of high or low maternal glycemic traits. Thus, we also examined the differences of maternal glycemic traits among maternal-fetal genotype combination groups, in addition to maternal and fetal genotype groups, for each SNP. The genotypes and maternal-fetal genotype combinations were coded as minor allele dosage number. For example, for SNP rs41457646, A is the minor allele and G is the major allele, so the genotypes and maternal-fetal genotype combinations were recoded as shown in Supplementary Table S4.

Analysis of covariance (ANCOVA) was used to adjust for maternal age at delivery, newborn sex, prepregnancy gravidity and prepregnancy parity. To examine whether the effect of maternal genotypes on maternal glycemic traits was independent of the effect of corresponding fetal genotypes, the effect of maternal genotypes on maternal glycemic traits was also adjusted by ANCOVA using corresponding fetal genotypes as categorical variables. For example, the effect of maternal rs12537573 genotypes on 24–28-week maternal FPI was adjusted for fetal rs12537573 genotypes. Similarly, the effect of fetal genotypes on maternal glycemic traits was also adjusted by ANCOVA using corresponding maternal genotypes as categorical variables. All *P* values were two sided.

## Electronic supplementary material

Supplementary Information
