# Supplementary material for: Effects of maternal and fetal LEP common variants on maternal glycemic traits in pregnancy
Source: Sci Rep. 2017 Dec 18;7:17710. doi: 10.1038/s41598-017-18117-z (PMC5735190; doi:10.1038/s41598-017-18117-z)
Supplement: Supplementary file 1 — Supplementary Information [file 41598_2017_18117_MOESM1_ESM.pdf]

Effects of maternal and fetal *LEP* common variants on maternal glycemic traits in pregnancy

Rong Lin[1,*], Hongfang Ju[2], Ziyu Yuan[3,4], Caicai Zhang[5], Liangliang Zeng[1], Yuantian Sun[1], Zhenyu Su[1] & Li Jin[3,4,6]

		Genotype	N	Mean(95% confidence interval)	*P*	*P*[a]	*P*[b]	*P*[c]
24-28-week maternal plasma glucose 1 hour after the consumption of a 50-g oral glucose load, mmol/l								
SNP 1	rs1349419_M	0	534	7.06(6.94-7.19)	0.023	0.027	0.411*	0.427*
		1	303	7.16(7.01-7.32)				
		2	44	7.68(7.21-8.15)				
SNP 2	rs7799039_M	0	533	7.07(6.95-7.20)	0.027	0.032	0.398*	0.403*
		1	304	7.15(6.99-7.31)				
		2	44	7.68(7.21-8.15)				
SNP 3	rs13228377_M	0	533	7.07(6.95-7.19)	0.026	0.031	0.382*	0.385*
		1	302	7.15(6.99-7.31)				
		2	44	7.68(7.21-8.15)				
SNP 10	rs11761556_M	0	474	7.01(6.88-7.14)	0.027	0.010	0.615*	0.506*
		1	353	7.25(7.10-7.41)				
		2	54	7.36(6.99-7.73)				
SNP 12	rs2060715_M	0	476	7.01(6.89-7.14)	0.034	0.013	0.615*	0.569*
		1	348	7.25(7.10-7.41)				
		2	57	7.35(6.98-7.71)				
SNP 9	rs41457646_F	0	480	7.18(7.05-7.31)	0.019	0.009[d]	0.072#	0.023#
		1	155	6.82(6.61-7.03)				
		2	16	7.30(6.75-7.85)				
SNP 9	rs41457646_MF	0	302	7.26(7.09-7.42)	0.037	0.030	0.213*	0.317*
		1	95	6.73(6.45-7.02)				
		2	61	7.07(6.71-7.42)				

		3	13	6.91(6.48-7.34)				
		4	2	7.35(0.36-14.34)				
24-28-week maternal fasting plasma insulin, pmol/l								
SNP 1	rs1349419_M	0	535	51.93(49.56-54.42)	0.048	0.063	0.037*	0.036*
		1	299	54.56(51.18-58.17)				
		2	44	44.00(37.12-52.17)				
SNP 4	rs2167270_M	0	564	52.01(49.71-54.42)	0.020	0.029	0.026*	0.023*
		1	274	54.82(51.24-58.64)				
		2	40	42.32(35.36-50.65)				
SNP 5	rs10244329_M	0	519	51.69(49.34-54.16)	0.049	0.062	0.037*	0.038*
		1	314	54.77(51.36-58.40)				
		2	45	44.65(37.74-52.83)				
SNP 6	rs11763517_M	0	548	51.56(49.29-53.94)	0.014	0.019	0.016*	0.016*
		1	287	55.46(51.81-59.37)				
		2	42	43.23(36.35-51.42)				
SNP 7	rs10954173_M	0	550	51.69(49.42-54.07)	0.008[d]	0.011	0.009[d]*	0.008[d]*
		1	288	55.36(51.72-59.25)				
		2	39	41.66(34.77-49.92)				
SNP 11	rs12537573_M	0	577	51.45(49.24-53.77)	0.006[d]	0.004[d]	0.003[d]*	0.004[d]*
		1	274	55.80(52.02-59.87)				
		2	27	40.25(32.91-49.22)				
SNP 1	rs1349419_F	0	374	53.89(50.73-57.24)	0.023	0.030	0.206#	0.229#
		1	250	50.85(47.28-54.69)				

		2	31	40.24(32.86-49.28)				
SNP 2	rs7799039_F	0	375	53.89(50.74-57.24)	0.024	0.032	0.215#	0.242#
		1	249	50.71(47.13-54.56)				
		2	32	40.55(33.31-49.36)				
SNP 3	rs13228377_F	0	378	54.01(50.87-57.35)	0.020	0.027	0.207#	0.234#
		1	246	50.50(46.91-54.37)				
		2	32	40.55(33.31-49.36)				
SNP 5	rs10244329_F	0	364	54.27(51.06-57.68)	0.006[d]	0.008[d]	0.087#	0.098#
		1	257	50.70(47.17-54.49)				
		2	35	39.21(32.45-47.38)				
SNP 6	rs11763517_F	0	397	54.17(51.18-57.34)	0.034	0.042	0.205#	0.199#
		1	229	49.66(45.83-53.8)				
		2	29	42.40(34.42-52.22)				
SNP 7	rs10954173_F	0	402	54.30(51.32-57.46)	0.019	0.024	0.123#	0.121#
		1	223	49.51(45.65-53.7)				
		2	30	41.62(33.92-51.07)				
SNP 1	rs1349419_MF	0	212	50.69(47.01-54.66)	0.039	0.050	0.392#	0.433#
		1	124	53.15(48.06-58.77)				
		2	94	52.42(46.70-58.84)				
		3	38	39.29(32.69-47.23)				
		4	4	37.71(19.78-71.90)				
SNP 2	rs7799039_MF	0	214	50.60(46.95-54.54)	0.044	0.056	0.108*	0.108*
		1	123	53.09(47.97-58.76)				

		2	93	52.43(46.65-58.92)				
		3	39	39.56(33.06-47.34)				
		4	4	37.71(19.78-71.90)				
SNP 5	rs10244329_MF	0	207	50.74(47.01-54.78)	0.016	0.021	0.208#	0.244#
		1	120	52.74(47.54-58.50)				
		2	102	53.20(47.75-59.27)				
		3	40	38.40(32.24-45.74)				
		4	4	37.71(19.78-71.90)				
SNP 6	rs11763517_MF	0	233	50.56(47.15-54.21)	0.037	0.046	0.236#	0.241#
		1	108	53.93(48.15-60.41)				
		2	91	51.89(46.04-58.48)				
		3	36	38.46(31.84-46.46)				
		4	3	45.31(26.82-76.54)				
SNP 7	rs10954173_MF	0	234	50.78(47.35-54.47)	0.012	0.017	0.125#	0.133#
		1	108	54.17(48.45-60.55)				
		2	90	51.78(45.89-58.43)				
		3	36	37.10(30.86-44.61)				
		4	3	45.31(26.82-76.54)				
SNP 11	rs12537573_MF	0	259	50.21(46.99-53.66)	0.042	0.058	0.624#	0.616#
		1	109	53.35(47.54-59.86)				
		2	79	52.57(46.05-60.00)				
		3	24	36.40(30.57-43.33)				
		4	2	40.69(11.56-143.16)				

HOMA2-β								
SNP 5	rs10244329_F	0	350	120.00(115.47-124.71)	0.048	0.052	0.202#	0.195#
		1	242	117.02(111.88-122.39)				
		2	33	102.34(91.81-114.08)				
SNP 7	rs10954173_F	0	387	120.57(116.31-125.00)	0.029	0.033	0.077#	0.077#
		1	208	115.41(109.86-121.24)				
		2	29	101.78(90.13-114.93)				
HOMA1-IR								
SNP 6	rs11763517_M	0	534	1.49(1.42-1.57)	0.040	0.050	0.048*	0.049*
		1	283	1.59(1.48-1.71)				
		2	40	1.25(1.04-1.51)				
SNP 7	rs10954173_M	0	536	1.49(1.42-1.57)	0.027	0.036	0.033*	0.034*
		1	284	1.59(1.48-1.71)				
		2	37	1.21(1.00-1.47)				
SNP 11	rs12537573_M	0	563	1.49(1.42-1.56)	0.016	0.013	0.010*	0.015*
		1	270	1.60(1.49-1.73)				
		2	25	1.15(0.92-1.43)				
SNP 5	rs10244329_F	0	360	1.57(1.47-1.67)	0.018	0.026	0.183#	0.216#
		1	254	1.47(1.37-1.59)				
		2	34	1.15(0.93-1.41)				
SNP 7	rs10954173_F	0	398	1.56(1.47-1.66)	0.050	0.064	0.238#	0.239#
		1	219	1.45(1.33-1.58)				
		2	30	1.21(0.97-1.50)				

SNP 5	rs10244329_MF	0	206	1.48(1.36-1.61)	0.039	0.053	0.152*	0.168*
		1	120	1.54(1.38-1.72)				
		2	102	1.53(1.37-1.72)				
		3	39	1.11(0.92-1.34)				
		4	3	1.19(0.66-2.12)				
SNP 7	rs10954173_MF	0	233	1.48(1.37-1.60)	0.042	0.057	0.310#	0.326#
		1	108	1.58(1.40-1.77)				
		2	90	1.48(1.31-1.69)				
		3	34	1.10(0.89-1.35)				
		4	3	1.19(0.66-2.12)				
HOMA2-IR								
SNP 4	rs2167270_M	0	541	0.97(0.93-1.01)	0.023	0.042	0.015*	0.018*
		1	265	1.01(0.95-1.06)				
		2	38	0.81(0.68-0.95)				
SNP 6	rs11763517_M	0	526	0.97(0.93-1.01)	0.033	0.057	0.013*	0.017*
		1	277	1.01(0.96-1.06)				
		2	40	0.82(0.70-0.97)				
SNP 7	rs10954173_M	0	528	0.97(0.93-1.01)	0.016	0.031	0.007[d]*	0.009[d]*
		1	278	1.01(0.95-1.06)				
		2	37	0.80(0.67-0.94)				
SNP 11	rs12537573_M	0	554	0.97(0.93-1.00)	0.004[d]	0.004[d]	1.40×10^{-4}[d]*	1.75×10^{-4}[d]*
		1	264	1.01(0.96-1.07)				
		2	26	0.74(0.61-0.90)				

SNP 3	rs13228377_F	0	363	1.00(0.95-1.05)	0.048	0.057	0.421#	0.414#
		1	232	0.96(0.90-1.02)				
		2	30	0.80(0.68-0.95)				
SNP 5	rs10244329_F	0	350	1.00(0.95-1.05)	0.010	0.012	0.113#	0.108#
		1	242	0.97(0.91-1.03)				
		2	33	0.77(0.65-0.91)				
SNP 7	rs10954173_F	0	387	1.00(0.95-1.05)	0.042	0.049	0.346#	0.347#
		1	208	0.95(0.89-1.02)				
		2	29	0.80(0.67-0.96)				
SNP 5	rs10244329_MF	0	198	0.96(0.89-1.02)	0.021	0.024	0.110*	0.135*
		1	114	0.99(0.91-1.08)				
		2	98	1.02(0.93-1.12)				
		3	36	0.79(0.69-0.91)				
		4	4	0.66(0.32-1.33)				
SNP 6	rs11763517_MF	0	223	0.95(0.90-1.01)	0.044	0.053	0.628#	0.649#
		1	103	1.01(0.92-1.10)				
		2	86	1.02(0.92-1.13)				
		3	33	0.78(0.67-0.91)				
		4	3	0.80(0.47-1.36)				
SNP 7	rs10954173_MF	0	224	0.96(0.90-1.02)	0.014	0.018	0.443#	0.417#
		1	103	1.01(0.93-1.11)				
		2	85	1.02(0.92-1.12)				
		3	33	0.75(0.64-0.87)				

		4	3	0.80(0.47-1.36)				
SNP 11	rs12537573_MF	0	247	0.95(0.90-1.01)	0.001[d]	0.001[d]	0.297#	0.281#
		1	104	1.01(0.92-1.10)				
		2	74	1.04(0.94-1.16)				
		3	23	0.68(0.57-0.80)				
		4	2	0.72(0.25-2.06)				

Supplementary Table S1. Relationship between *LEP* common variants and glycemic traits. Values are shown as arithmetic mean (95% confidence interval) for plasma glucose and geometric mean (95% confidence interval) for other variables, as well as only for polymorphisms with $P<0.05$ in analysis of variance. [a]Adjusted for maternal age at delivery, newborn sex, prepregnancy gravidity and prepregnancy parity by analysis of covariance. [b]Adjusted for corresponding maternal or fetal variants by analysis of covariance. Adjustments for corresponding fetal variants were marked with *, and adjustments for corresponding maternal variants were marked with #. [c]Adjusted for corresponding maternal or fetal variants, maternal age at delivery, newborn sex, prepregnancy gravidity and prepregnancy parity by analysis of covariance. [d]$P<0.01$. M: maternal genotypes, F: fetal genotypes, MF: maternal and fetal genotype combinations, 0: genotype (combination) with zero copies of the minor allele, 1: genotype (combination) with 1 copy of the minor allele, 2: genotype (combination) with 2 copies of the minor allele, 3: genotype combination with 3 copies of the minor allele, 4: genotype combination with 4 copies of the minor allele. For example, for SNP rs41457646, A is the minor allele and G is the major allele, 1 for rs41457646_M indicates the maternal genotype is 'GA', 0 for rs41457646_F indicates the fetal genotype is 'GG', and 4 for rs41457646_MF indicates the genotype combination where the maternal genotype is 'AA' and the fetal genotype is also 'AA'.

Genotype		N	Geometric mean (95% confidence interval)	***P***		
**Fetal**	**Maternal**					
24-28-week maternal fasting plasma insulin, pmol/l						
AA	AA	207	50.74(47.01-54.78)	**0.021**	0.486	0.974
	AT	55	55.36(46.93-65.31)	**0.008**	0.670	0.383
AT	AA	65	50.62(44.26-57.88)	0.035	0.576	**1 (reference)**
	AT	102	53.20(47.75-59.27)	0.011	**1 (reference)**	0.576
	TT	19	37.20(28.27-48.95)	**1 (reference)**	0.011	0.035
TT	AT	21	39.53(30.94-50.50)	0.732	0.027	0.079
	TT	4	37.71(19.78-71.90)	0.965	0.228	0.308

Supplementary Table S2. Relationship between *LEP* SNP rs10244329 and 24-28-week maternal fasting plasma insulin.

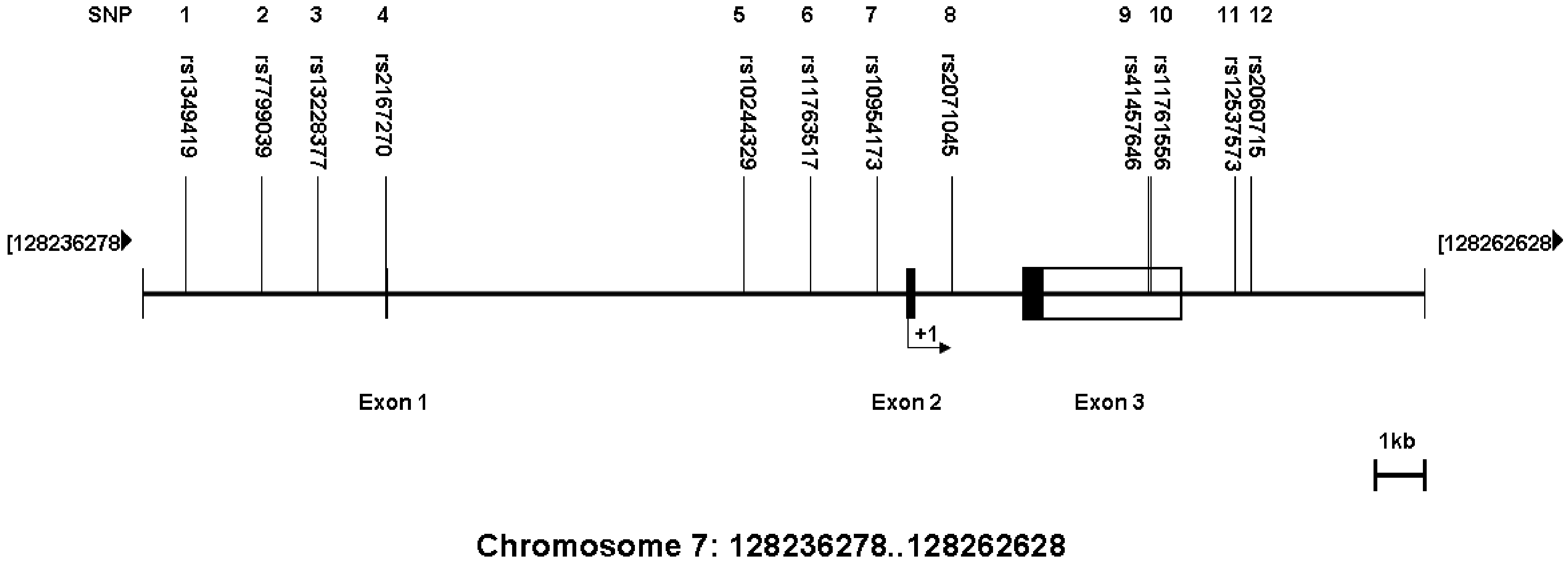

Supplementary Figure S1. Map of *LEP* on chromosome 7q32.1 (chromosome 7: 128236278..128262628 26.35 kbp).

This track displays transcript from the Ensembl release 81.38 annotation of the NCBI 38.p3 assembly of the human genome. Black boxes represent exons and white boxes represent 5'- and 3'-untranslated regions. The translation initiation site is indicated by the notation +1. The scale bar indicated a chromosomal distance of 1.0 kb.

No.	SNPs	Chromosome position[a]	Minor/ Major allele	Location in gene region	Call rate	HWE (*P* value) (maternal)	HWE (*P* value) (fetal)	MAF (maternal)	MAF (fetal)	MAF[b]				
										EAS	EUR	AFR	AMR	SAS
1	rs1349419[c]	Chr 7:128237160	A/G	5'-flanking	0.999	0.737	0.127	0.221	0.235	0.267	0.448	0.965	0.512	0.423
2	rs7799039	Chr 7:128238730	G/A	5'-flanking	1.000	0.769	0.177	0.221	0.235	0.269	0.558	0.968	0.591	0.486
3	rs13228377[c]	Chr 7:128239891	G/A	5'-flanking	0.999	0.719	0.208	0.221	0.233	0.266	0.483	0.225	0.467	0.366
4	rs2167270	Chr 7:128241296	A/G	5'-UTR	0.999	0.329	0.445	0.200	0.202	0.197	0.370	0.443	0.406	0.275
5	rs10244329	Chr 7:128248636	T/A	intron 1	1.000	0.778	0.201	0.231	0.246	0.281	0.514	0.543	0.523	0.465
6	rs11763517	Chr 7:128250009	C/T	intron 1	0.999	0.579	0.563	0.211	0.217	0.213	0.505	0.156	0.483	0.340
7	rs10954173[c]	Chr 7:128251387	A/G	intron 1	0.999	0.831	0.869	0.207	0.214	0.207	0.382	0.151	0.388	0.317
8	rs2071045	Chr 7:128252927	T/C	intron 2	1.000	0.175	0.698	0.415	0.420	0.439	0.768	0.988	0.732	0.823
9	rs41457646	Chr 7:128256968	A/G	3'-UTR	0.999	0.608	0.310	0.135	0.145	0.110	0.129	0.006	0.117	0.270
10	rs11761556	Chr 7:128257016	C/A	3'-UTR	0.999	0.323	0.445	0.262	0.257	0.289	0.464	0.882	0.522	0.427
11	rs12537573	Chr 7:128258747	G/A	3'-flanking	1.000	0.462	0.981	0.187	0.186	0.192	0.390	0.101	0.392	0.321
12	rs2060715[c]	Chr 7:128259076	A/G	3'-flanking	0.999	0.601	0.474	0.263	0.259	0.291	0.469	0.881	0.522	0.469

Supplementary Table S3. Primary information for genotyped SNPs. [a] Positions according to the human genome assembly Build 38. [b] available from the 1000 Genomes Project, based on GRCh38. [c] Candidate SNPs. EAS, East Asian; EUR, European; AFR, African; AMR, Ad Mixed American; SAS, South Asian.

Maternal genotype	Code for maternal genotype	Fetal genotype	Code for fetal genotype	Maternal-fetal genotype combination	Code for maternal-fetal genotype combination
GG	0	GG	0	GG-GG	0
GG	0	GA	1	GG-GA	1
GA	1	GG	0	GA-GG	1
GA	1	GA	1	GA-GA	2
GA	1	AA	2	GA-AA	3
AA	2	GA	1	AA-GA	3
AA	2	AA	2	AA-AA	4

Supplementary Table S4. Codes for the genotypes and maternal-fetal genotype combinations of SNP rs41457646. For SNP rs41457646, A is the minor allele and G is the major allele. The genotypes and maternal-fetal genotype combinations were coded as minor allele dosage number.
